# Acromegaly is associated with an increased incidence of primary malignant tumors: data from a national study in Sweden

**DOI:** 10.1210/clinem/dgag137

**Published:** 2026-03-30

**Authors:** Erika Tsatsaris, Jonas Robèrt, Pia Burman, Katarina Berinder, Lorenza Bonelli, Per Dahlqvist, Charlotte Höybye, Oskar Ragnarsson, Konstantina Vouzouneraki, Anna-Karin Åkerman, Bertil Ekman, Britt Edén Engström

**Affiliations:** Department of Medical Sciences, Endocrinology and Mineral Metabolism, Uppsala University Hospital, Uppsala University, Uppsala SE-751 85, Sweden; Departments of Endocrinology in Linköping and Norrköping, and Department of Health, Medicine and Caring Sciences, Linköping University, Linköping SE-581 83, Sweden; Department of Endocrinology, Skåne University Hospital Malmö, Lund University, Malmö SE-205 02, Sweden; Department of Endocrinology, Karolinska University Hospital, Stockholm SE-171 76, Sweden; Department of Molecular Medicine and Surgery, Karolinska Institutet, Stockholm SE-171 76, Sweden; Department of Endocrinology, Skåne University Hospital Malmö, Lund University, Malmö SE-205 02, Sweden; Department of Public Health and Clinical Medicine, Umeå University, Umeå SE-901 87, Sweden; Department of Endocrinology, Karolinska University Hospital, Stockholm SE-171 76, Sweden; Department of Molecular Medicine and Surgery, Karolinska Institutet, Stockholm SE-171 76, Sweden; Department of Endocrinology, Sahlgrenska University Hospital, Gothenburg SE-413 45, Sweden; Department of Internal Medicine and Clinical Nutrition, Institute of Medicine at Sahlgrenska Academy, University of Gothenburg, Gothenburg SE-413 45, Sweden; Wallenberg Center for Molecular and Translational Medicine, University of Gothenburg, Gothenburg SE-413 45, Sweden; Department of Public Health and Clinical Medicine, Umeå University, Umeå SE-901 87, Sweden; Department of Internal Medicine, Örebro University Hospital, Örebro SE-701 82, Sweden; Faculty of Health and Medical Sciences, Örebro University, Örebro SE-701 82, Sweden; Departments of Endocrinology in Linköping and Norrköping, and Department of Health, Medicine and Caring Sciences, Linköping University, Linköping SE-581 83, Sweden; Department of Medical Sciences, Endocrinology and Mineral Metabolism, Uppsala University Hospital, Uppsala University, Uppsala SE-751 85, Sweden

**Keywords:** acromegaly, cancer, mortality, patient register

## Abstract

**Context:**

GH excess and elevated IGF-1 in acromegaly are considered to promote cancer development.

**Objective:**

To investigate cancer incidence and outcome in patients with acromegaly in relation to biochemical control.

**Methods:**

Matched cohort study in patients with acromegaly diagnosed from 1991 to 2018 and 10 controls per case from the Swedish population. Cancer diagnoses and fatalities were obtained from the Swedish Pituitary and National Patient Registers. Adjusted hazard ratio (HR) and 95% confidence intervals (CIs) for cancer incidence and death were estimated using a Cox proportional hazard regression model adjusted for age, sex, and comorbidity.

**Results:**

We included 1035 patients with acromegaly (49.5% female; median age 52.0 years) and 10 261 matched controls. Patients had higher adjusted HR (95% CI) for all cancer (1.28, 1.11-1.49), colorectal cancer (1.84, 1.28-2.64), lung cancer (1.95, 1.22-3.11), hematologic cancer (1.68, 1.03-2.73), and breast cancer in women (1.46, 1.02-2.11) from 5 years before acromegaly diagnosis. Second cancers after diagnosis tended to be increased (1.54, 0.98-2.44). Death from cancer was only significantly elevated in patients 40 to 60 years of age (1.48, 1.19-1.85). Patients with persistently elevated IGF-1 had a higher overall mortality rate (1.50, 1.10-2.01) but no increase in cancer incidence or cancer-related mortality compared to biochemically controlled patients.

**Conclusion:**

This nationwide, matched cohort study showed an increased risk of cancer in patients with acromegaly, underscoring the importance of vigilance for early signs of cancer after acromegaly diagnosis. Biochemical control had minor effects on increased cancer development, indicating an effect beyond GH hypersecretion.

With an annual incidence of 4 cases per million (1-3) and a prevalence of 2.8 to 13.7 per 100 000 subjects (4), acromegaly is considered a rare condition. It is characterized by chronic excess of GH resulting in elevated levels of IGF-1, and its diagnosis usually takes several years to be confirmed (5). The sustained overproduction of GH and IGF-1 in acromegaly is associated with multiple comorbidities such as heart failure, diabetes, sleep apnea, cerebrovascular disease (6-8), and increased mortality due to cardiovascular events (9-11).

Experimental studies have suggested that GH and IGF-1 excess are associated with cell proliferation, differentiation, and angiogenesis, which potentially lead to cancer promotion (12, 13). However, it is still unclear whether acromegaly is linked to increased cancer incidence or cancer-related mortality.

Some studies present a higher cancer risk for all types of malignancy compared to the general population (14-17), while others report a higher risk for specific cancer types, including colorectal (18, 19), thyroid (20, 21), breast (22, 23), prostate (24), and renal cancer (19). Other large epidemiological studies did not find a significant difference in cancer risk compared to the general population (25-27). Moreover, available data for cancer-related mortality in patients with acromegaly show conflicting results (11, 22, 28, 29).

These inconsistencies may be related to several limitations in the design of the available studies. Single-center studies present higher cancer incidence in patients with acromegaly, suggesting a potential selection and sample bias (14). Additionally, a small sample size for adjusting basic confounding factors, heterogeneity in both the sample group and the general population, varying disease activity in the patients with acromegaly, and better therapeutic methods for acromegaly leading to a prolonged life expectancy may contribute to these discrepancies (22, 30).

The aim of our current study was to estimate the relative hazard for cancer and cancer-related mortality in a national cohort of patients with acromegaly compared to a matched control group from the general population and to investigate whether this risk is related to the biochemical control of acromegaly.

## Methods

### Study design and data sources

This observational study was conducted with data from several different nationwide Swedish registries. The National Patient Register (NPR) and the Swedish Pituitary Register (SPR) were used to identify subjects with acromegaly. The SPR was established in 1991 and is financially supported by the Swedish government as a national quality register. SPR includes information regarding pituitary radiology, visual disturbances, laboratory results, surgery, radiation therapy, medical treatment for the pituitary tumor, hormonal substitution therapy, and outcome, including patient-reported outcome measures. The NPR covers all of Sweden since 1987 and contains International Classification of Diseases (ICD) [9th revision (ICD-9) and 10th revision (ICD-10)] codes of diagnoses related to hospitalizations and outpatient visits as well as data on Classification of Care Measures regarding surgical procedures.

Information on malignant tumors was retrieved from the Swedish Cancer Register. The Swedish Cancer Register was initiated in 1958 and provides nationwide coverage (>96%) (31). Data on mortality (general and cancer-related) were acquired from the Swedish National Cause of Death Register, which includes information on the time and cause of death of every deceased citizen in Sweden since 1952. The Swedish Prescribed Drug Register contains data about all dispensed medication, starting from July 2005. Cross-links between all registries named previously were established using the personal identification number, which is unique for every Swedish resident.

### Study population

Patients from 18 years of age registered with acromegaly in the SPR and those with ICD codes 253A (ICD-9) and E220 (ICD-10) in the NPR during the study period (January 1, 1991, to December 31, 2018) were initially included. To minimize the risk of unvalidated acromegaly diagnosis, we applied an algorithm, as described in our previous study (3). All patients were followed from the diagnosis of acromegaly until the end of the study period, migration, loss to follow-up, or death. The primary index date in the study was set to the date of acromegaly diagnosis.

Due to the long diagnostic delay in acromegaly, we also collected data on the first occurrence of malignant tumor diagnosis from 5 years before the index date of acromegaly diagnosis until the end of the study period in both patients with acromegaly and control subjects.

### Control population

For each patient with acromegaly, 10 controls were included from the general population matched for sex, age, and municipality of residence using Statistics Sweden, the official government body for general population statistics in Sweden.

### Diagnostic codes

Cancer diagnoses in patients and controls were identified in the Swedish Cancer Register using the following ICD codes:

- Colorectal cancer (ICD-9: 153-154; ICD-10: C18-C21)
- Lung cancer (ICD-9: 162; ICD-10: C34)
- Hematological cancer (ICD-9: 200-208; ICD-10: C81-C96)
- Breast cancer (ICD-9: 174-175; ICD-10: C50)
- Urogenital cancer (ICD-9: 179-189; ICD-10: C51-58, C60-68)
- Prostate cancer (ICD-9: 185; ICD-10: C61)
- Skin cancer (ICD-9: 173; ICD-10: C44)
- Thyroid cancer (ICD-9: 193; ICD-10: C73)
- Other types of cancer (excluding the previously described types) (ICD-9: 140-208; ICD-10: C00-C97)

Other diagnoses were defined in the NPR using the following ICD codes:

- Diabetes (ICD-9: 250; ICD-10: E10-E11)
- Hypertension (ICD-9: 401-405; ICD-10: I10.9)
- Cardiovascular disease (ICD-9: 410-414; ICD-10: I20-I25)
- Cerebrovascular disease (ICD-9: 430-438; ICD-10: I60-I69)

Pituitary surgery as well as radiation therapy of the pituitary were identified by the combined use of SPR and a specific code in the NPR unique for each type of treatment:

- Pituitary surgery (AAE10)
- Radiation therapy (AAG50)

### Registered variables

Biochemical control (with or without medical therapy) was defined as serum IGF-1 values within the age-specific reference range predefined by the respective laboratory, which means that upper limit of normal for aged-adjusted IGF-1 levels was used as cutoff. A pituitary tumor with the largest diameter ≥10 mm was defined as macroadenoma, whereas a tumor with the largest diameter <10 mm was defined as microadenoma. Pituitary hormone deficiency was defined as deficiency in at least 1 pituitary hormone defined according to reference intervals for methods used at the time of testing and in accordance with current national and international guidelines.

### Ethics approval

This study and the data collection it entailed have been approved by the Regional Ethics Review Board, Uppsala, Sweden (No. 2017/475). The SPR is approved by the Ethics Review Board, Karolinska Institute, Stockholm, Sweden (No. 2003/515/03 and No. 2012/915-32). Patients in the SPR must be informed and given the option to opt out of the register at any time. No informed consent is required for registration in national quality registers. This study was performed in accordance with the Declaration of Helsinki 1964 and its later amendments.

### Statistical analysis

Baseline characteristics were summarized with median and quartiles for continuous data and with count and proportion for categorical data. Two separate index dates were defined: at acromegaly diagnosis and 5 years before acromegaly diagnosis. For each analysis, the index date was the same for the patient with acromegaly and the corresponding matched control. We censored for the first of emigration, end of study (December 31, 2018), or death. Follow-up was defined as time from index date to the date of an event or censoring. We used Kaplan-Meier curves to describe the cumulative event rates of acromegaly patient-specific endpoints among acromegaly patients as well as the cumulative event rate of all the main endpoints stratified for patients with acromegaly and controls. For time-dependent covariates, we used Simon-Makuch curves.

A Cox proportional hazard regression model was used to estimate adjusted hazard ratios (HRs) and 95% confidence intervals (CIs) for the effect of having acromegaly in the development of cancer diagnoses (specified previously) and death (all cause and cancer-specific). We adjusted for age, sex, and comorbidities as a way of controlling for confounding and to control for the matching on sex and age. Municipality as a matching factor was not handled in the analysis.

To assess the effect of biochemical control, pituitary surgery, pituitary radiation therapy, and number of pituitary hormone deficiencies (1 or >1), we included each of them separately as time-dependent covariates in a Cox proportional hazard regression model using only the patients with acromegaly and adjusted for the same variables as stated previously. Further, to examine whether the effect on the outcomes of having acromegaly could be attributed to failing to reach biochemical control, we performed a post hoc time-dependent Cox proportional hazard regression model, adjusted for the same variables as stated previously, using both the patients with acromegaly and controls. The controls would then stay in the state of being controls throughout, whereas patients with acromegaly could transition between biochemical control and nonbiochemical control over the follow-up. For all models, a likelihood ratio test was used and *P* < .05 was considered statistically significant. No multiplicity adjustments and no sensitivity analyses were made. There were no missing data for any of the covariates.

Subgroup analyses in the whole cohort were performed for all primary endpoints by stratifying the study population based on age (18-39, 40-60, and >60 years), diabetes (no/yes), hypertension (no/yes), cardiovascular disease (no/yes), cerebrovascular disease (no/yes), and sex (female/male).

All analyses were performed with R version 4.4.1 and all inference using the survival package version 3.6.4.

## Results

### Descriptive statistics

**Figure 1:**
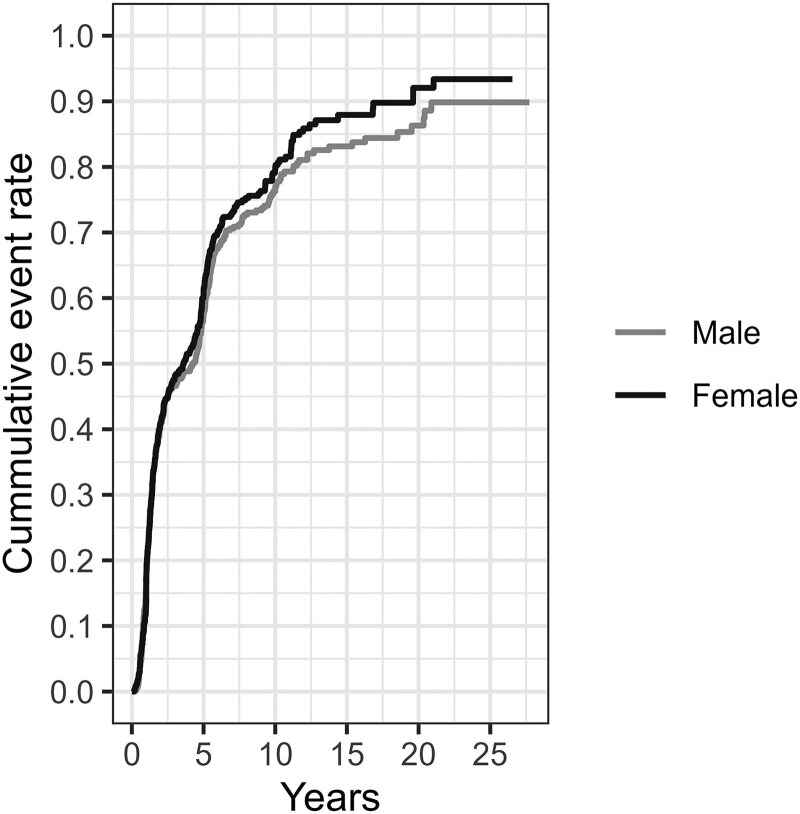
Kaplan-Meier curve describing the cumulative event rate from time of acromegaly diagnosis to first biochemical control during the study.

A total of 1035 patients with acromegaly and 10 261 matched controls were included. Table 1 presents the characteristics of the acromegaly and control groups at the time of diagnosis. Overall, median (interquartile range) age was 52.0 (40.0-62.5) years (49.5% female) in the acromegaly group compared to 52.0 (40.0-63.0) years (49.3% female) in the control group. The small differences found between patients with acromegaly and controls were due to the fact that it was not always possible to obtain 10 matched controls in smaller municipalities. Diabetes (8.0% vs 3.3%) and hypertension (14.2% vs 5.7%) were more frequent in the acromegaly group compared to controls, while respective rates of cardiovascular disease and cerebrovascular disease were similar across the groups. Overall, median (interquartile range) follow-up duration was similar across the groups: 10.8 (5.2-17.5) years in the acromegaly group vs 11.0 (5.3-17.8) years in the control group. Pituitary tumor characteristics and treatment of patients with acromegaly are shown in Table 2. The cumulative event rate of biochemical control is shown in Fig. 1. There was no difference between females and males in the rate of biochemical control.

**Table 1:** Characteristics at diagnosis of patients with acromegaly vs controls and follow-up duration

Characteristic	Acromegaly (n = 1035)	Controls (n = 10 261)
Age, years (IQR)	52.0 (40.0-62.5)	52.0 (40.0-63.0)
Female (%)	512 (49.5)	5054 (49.3)
Male (%)	523 (50.5)	5207 (50.7)
Diabetes (%)	83 (8.0)	336 (3.3)
Hypertension (%)	147 (14.2)	588 (5.7)
Cardiovascular disease (%)	45 (4.3)	425 (4.1)
Cerebrovascular disease (%)	28 (2.7)	243 (2.4)
Follow-up, years (IQR)	10.8 (5.2-17.5)	11.0 (5.3-17.8)

Data are n (percent) or median (IQR).

Abbreviations: IQR, interquartile range.

**Table 2:** Tumor size, pituitary deficiencies, and treatment modalities in patients with acromegaly

Characteristic	Acromegaly (n = 1035)
Tumor size,*^a^* (%)	
Microadenoma (<10 mm)	245 (27.3)
Macroadenoma (≥10 mm)	647 (72.1)
Not visible	5 (0.6)
Tumor volume,*^b^* mm^3^ (IQR)	864 (255-3028)
Pituitary deficiency (%)	
1 deficiency at baseline	183 (17.7)
1 deficiency overall	340 (32.9)
≥2 deficiencies at baseline	57 (5.5)
≥2 deficiencies overall	169 (16.3)
Treatment modality (%)	
Pituitary surgery	831 (80.3)
Radiation therapy overall	140 (13.5)
Fractionated radiation therapy (photons)	34 (3.3)
Fractionated radiation therapy (protons)	8 (0.8)
Gamma knife therapy	98 (9.5)
Medical therapy derived from SPR*^c^* (%)	
Medical therapy overall	289 (27.9)
Dopamine agonist	81 (7.8)
Somatostatin analogue	237 (22.9)
GH receptor antagonist	47 (4.5)
Medical therapy derived from NPDR*^d^* (%)	
Dopamine agonist	57 (10.9)
Somatostatin analogue	212 (40.6)
GH receptor antagonist	54 (10.3)

Data are n (percent) or median (IQR).

Abbreviations: IQR, interquartile range; NPDR, National Patient Drug Registry; SPR, Swedish Pituitary Register.

^*a*^Data not reported for 138 patients.

^*b*^Data not reported for 199 patients.

^*c*^Data derived from the SPR for drugs registered from 1991.

^*d*^Data derived from the NPR for drugs registered from 2005.

### Cancer incidence

**Figure 2:**
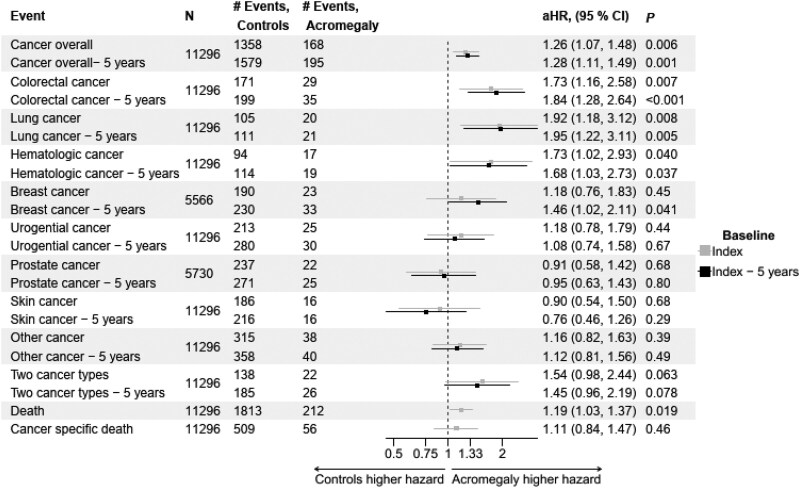
Cox proportional hazard regression analysis on the risk for the development of cancer and death in patients with acromegaly and in controls from acromegaly diagnosis and from 5 years before diagnosis until study end adjusted for sex, age, and comorbidities. Breast cancer is reported from 512 women with acromegaly and 5054 controls. Prostate cancer is reported from 523 men with acromegaly and 5207 controls. Cancer subtypes in the patients from diagnosis were colorectal cancer (colon [n = 25], rectum [n = 3], and rectosigmoid junction [n = 1]); lung cancer (adenocarcinoma [n = 13], neuroendocrine malignant tumor [n = 5], and squamous cell carcinoma [n = 3]); and hematologic cancer (multiple myeloma [n = 5], follicular lymphoma [n = 4], nonfollicular lymphoma [n = 3], B-cell lymphoma [n = 2], chronic myeloid leukemia [n = 2], and acute lymphatic leukemia [n = 1]). Abbreviations: aHR, adjusted hazard ratio; CI, confidence interval.

**Figure 3:**
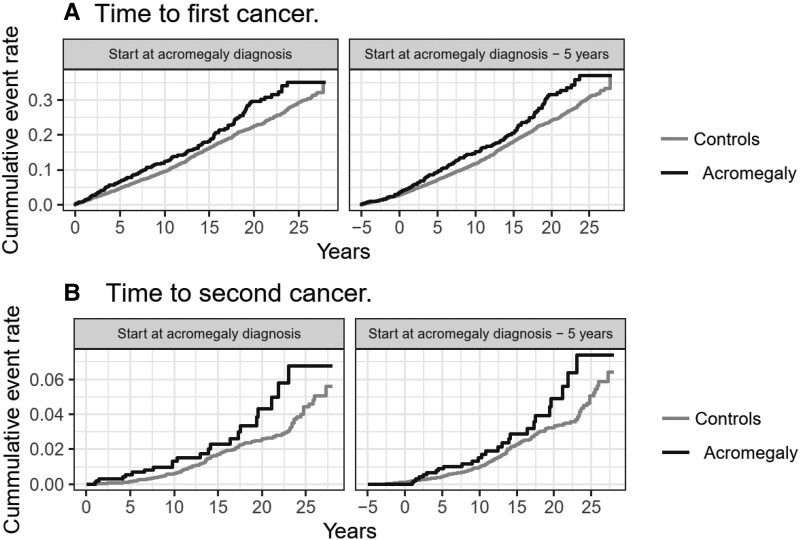
Kaplan-Meier curves describing the cumulative event rates to the occurrence of (A) first and (B) second primary cancer of all types in patients with acromegaly and controls from acromegaly diagnosis and from 5 years before acromegaly diagnosis.

**Figure 4:**
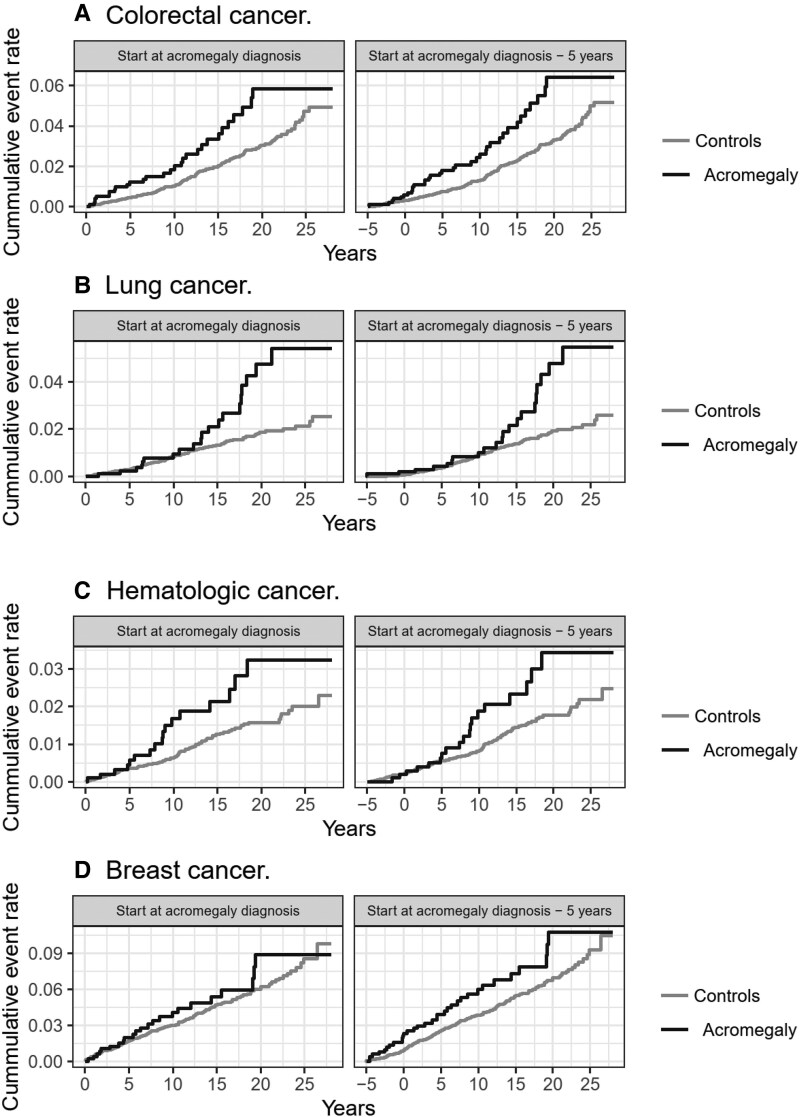
Kaplan-Meier curves describing the cumulative event rates to the occurrence of first (A) colorectal cancer, (B) lung cancer, (C) hematologic cancer, and (D) female breast cancer in patients with acromegaly and controls from acromegaly diagnosis and from 5 years before acromegaly diagnosis.

Cancer of all causes (cancer overall) was diagnosed in 16.2% (n = 168) of the patients with acromegaly and in 13.2% (n = 1358) of the controls. A Cox regression analysis adjusted for age, sex, and comorbidities (diabetes mellitus, hypertension, cardiovascular disease, and cerebrovascular disease) showed an increased adjusted HR for developing cancer overall and colorectal, lung, and hematologic cancer in the patients with acromegaly compared to controls from the primary index date. When including the 5 years before acromegaly diagnosis until study end, the adjusted HR was increased for the same cancer types and also became significant for breast cancer in women (Fig. 2). Twenty-two patients (2.1%) from an acromegaly diagnosis and 26 patients (2.5%) from 5 years before an acromegaly diagnosis had a subsequent second cancer. Corresponding numbers for the controls were 138 (1.3%) and 185 (1.8%), respectively (Fig. 2). Table 3 details the different cancer types among the 22 patients from diagnosis who had a subsequent second cancer. Kaplan-Meier curves describing cumulative event rates are shown for cancer overall (Fig. 3A), second cancer (Fig. 3B), colorectal cancer (Fig. 4A), lung cancer (Fig. 4B), hematologic cancer (Fig. 4C), and female breast cancer (Fig. 4D) in patients with acromegaly and controls.

**Table 3:** Cancer types among the 22 patients with acromegaly who developed a secondary cancer, including 2 patients who developed a third cancer

First cancer	Sex and age*^a^* (years)	Time from first to second cancer (months)	Second cancer	Time from second to third cancer (months)	Third cancer
Prostate	M (65)	3	Skin		
	M (74)	45	Lung		
	M (60)	113	Hematologic		
	M (49)	127	Urogenital		
Colorectal	M (72)	42	Skin		
	M (56)	6	Skin		
	F (53)	51	Melanoma		
	M (69)	55	Hematologic		
Breast	F (49)	8	Colorectal		
	F (68)	51	Urogenital		
	F (66)	72	Hematologic		
Urogenital	M (55)	7	Lung		
	M (51)	71	Prostate	2	Lung
Hematologic	F (67)	10	Urogenital		
	F (45)	194	Pancreas		
Skin	M (68)	13	Prostate		
	F (70)	31	Urogenital		
Lung	M (51)	0	Urogenital		
	M (51)	54	Prostate	13	Liver
Thyroid	F (58)	13	Breast		
Melanoma	F (71)	7	Colorectal		
Pancreas	M (57)	68	Urogenital		

^*a*^Age at acromegaly diagnosis.

Abbreviations: F, female; M, male.

Adjusted HR (95% CI) showed no association between cancer overall in the patients with acromegaly and the presence of diabetes (1.13, 0.59-2.17), hypertension (1.22, 0.79-1.87), cardiovascular disease (1.09, 0.52-2.26), or cerebrovascular disease (1.02, 0.35-2.94).

A Cox regression model with time-dependent covariates adjusted for age, sex, and comorbidities showed a borderline significant adjusted HR (95% CI) for cancer overall in patients not biochemically controlled (1.24, 0.97-1.59) compared to controls.

### Effect of time-dependent covariates

**Figure 5:**
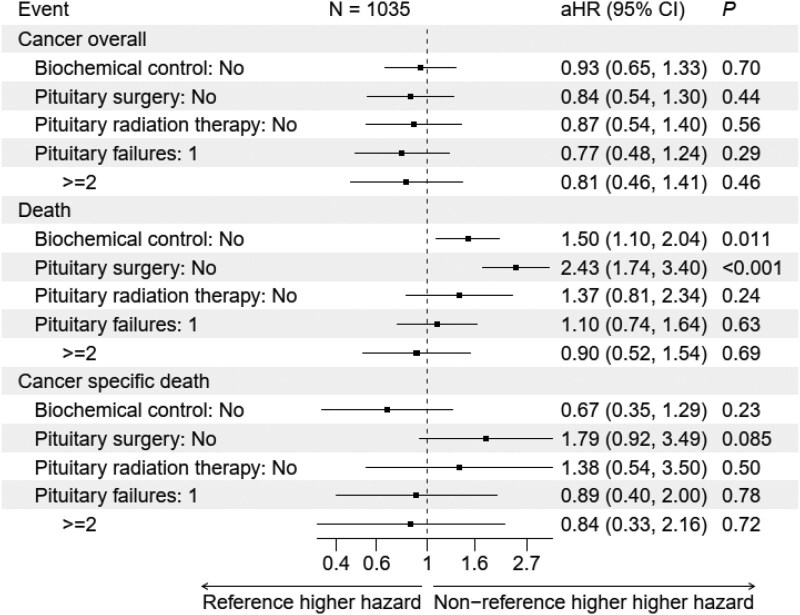
Cox proportional hazard regression analysis for patients with acromegaly with time-dependent covariates adjusted to sex, age, and comorbidities with respect to cancer overall, death, and cancer-specific death. Reference levels are no pituitary failure, pituitary surgery, pituitary radiation therapy, and biochemical control. Abbreviations: aHR, adjusted hazard ratio; CI, confidence interval.

**Figure 6:**
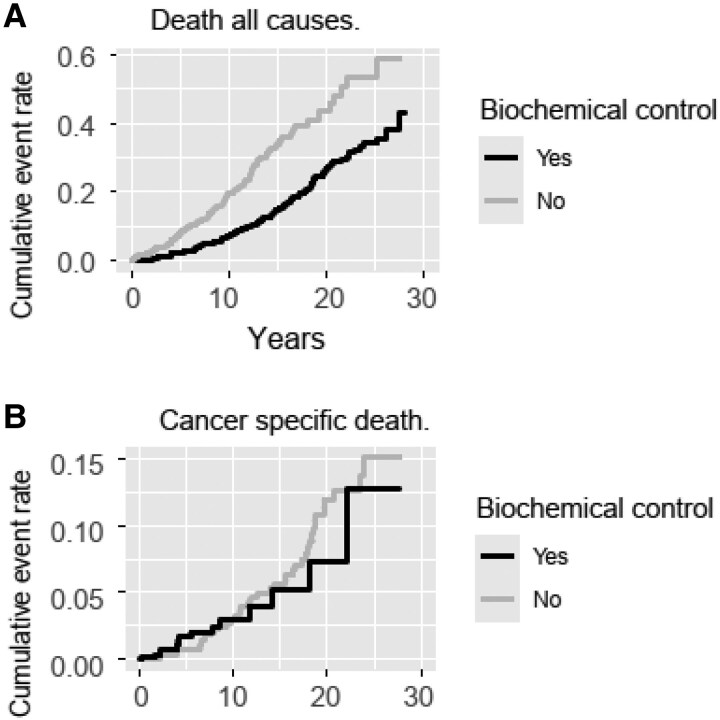
Simon-Makuch curves describing the cumulative event rates to the occurrence of (A) death from any cause and (B) cancer-specific death. Death from any cause was higher in patients not biochemically controlled compared to those biochemically controlled, while there was no difference for cancer-specific death.

Biochemical control, pituitary surgery, radiation therapy, and hypopituitarism had no major effect on cancer overall in patients with acromegaly (Fig. 5). Patients with acromegaly who did not have biochemical control indicated by elevated IGF-1 levels had an increased risk of death by any cause compared to those who were biochemically controlled (Fig. 6A), but there was no difference between these groups for cancer-specific death (Fig. 6B).

### Mortality

**Figure 7:**
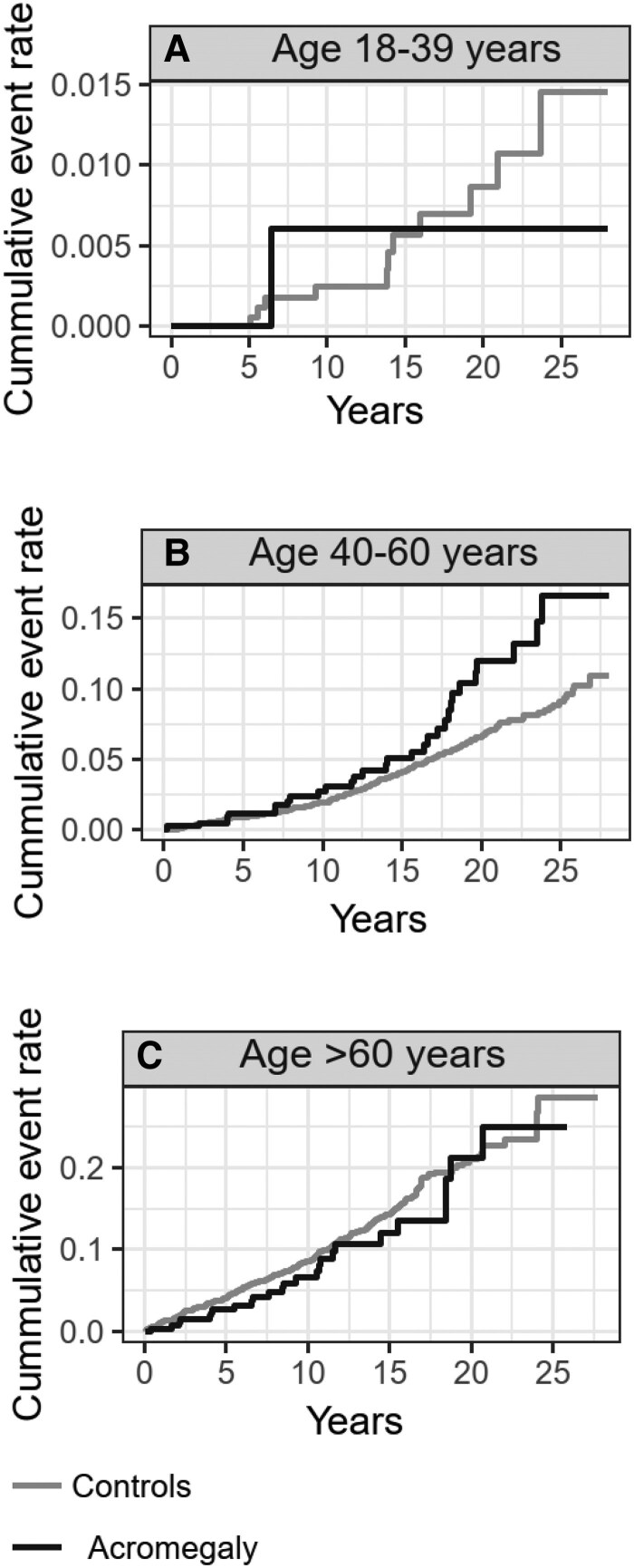
Kaplan-Meier curves describing the cumulative event rates to the occurrence of cancer-specific death according to age at start of acromegaly diagnosis: (A) age 18 to 39 years, (B) age 40 to 60 years, and (C) > 60 years. Adjusted hazards ratio (95% confidence interval) for cancer-specific death was increased in patients 40 to 60 years of age (1.54, 1.04-2.28) but not in those 18 to 39 years of age (too few events) or >60 years of age (0.81, 0.54-1.23).

Adjusted HR (95% CI) for overall mortality was increased in patients with acromegaly compared to controls (1.19, 1.03-1.37; *P* = .019) (Fig. 2). Additional analysis revealed that this was significantly due to cardiovascular disease (1.76, 1.22-2.53; *P* = .002) but not to diabetes (1.14, 0.75-1.75), hypertension (1.11, 0.79-1.58), or cerebrovascular disease (0.82, 0.45-1.50). Adjusted HR (95% CI) for cancer-specific death was increased in patients with acromegaly 40 to 60 years of age (1.54, 1.04-2.28; *P* = .031) but not in those >60 years (0.81, 0.54-1.23), while there were too few cancer-related deaths in those <40 years to permit analysis (Fig. 7A-7C). Adjusted HR (95% CI) revealed a higher rate for overall mortality among patients with persistently elevated IGF-1 levels compared to controls (1.40, 1.15-1.71, *P* < .001) but not compared to cancer-specific death (0.75, 0.46-1.25).

## Discussion

In this comprehensive, nationwide, matched cohort study of patients with acromegaly, we report an increased incidence of cancer overall, primarily related to colorectal, lung, hematologic, and breast cancers. The increased cancer incidence was already detected before the diagnosis of acromegaly. This underlines the importance of diagnosing acromegaly earlier to attenuate the possible effects of increased GH/IGF-1 levels on the increased incidence of cancer (4, 32, 33).

We found that persistent elevated IGF-1 levels did not further increase the overall cancer incidence compared to patients with biochemical control. This contrasts with a recently published study of 598 patients with acromegaly where cumulative exposure to IGF-1 excess was reported to be a predictor of cancer (34). It is conceivable that long-standing GH and IGF-1 hypersecretion preceding the diagnosis of acromegaly could promote cancer development even after achievement of biochemical control. Although GH and IGF-1 hypersecretion may have a role in the progression of malignancy, a combination of a strong family history of cancer and unfavorable environmental factors should also be considered (35). Already in a study from 2008, it was hypothesized that acromegaly, independent of hormone secretion, is a disease that heralds genetic and/or epigenetic alterations predisposing to increased cancer risk (36).

Another interesting observation in our study was the tendency for a higher adjusted HR for a second cancer type. The subtype distribution of the subsequent cancer was generally not in accordance with known hereditary endocrine cancer syndromes (37, 38). In a study of 394 patients with acromegaly (39), second primary tumors were described in nine of the 63 patients who had a primary malignant tumor. As in our study, no relationship between circulating GH and IGF-1 levels and cancer was found. Taken together, the lack of a clear relation between IGF-1 levels and cancer in combination with a higher incidence of second tumors may support the theory of other underlying genetic factors.

The increased incidence of overall cancer in patients with acromegaly in our study is in line with other nationwide studies (14, 19, 40, 41), including a recently published national study of 470 patients, which revealed a higher prevalence of malignant solid tumors compared to controls (42). However, the types of more prevalent cancers vary across different studies, and since Standardized Incidence Ratio (SIR) and HRs aim to describe the same thing, our estimate would still be comparable to similar studies that have only estimated a SIR. The present study cohort and that of Esposito et al (40) are partly overlapping. While the Esposito study, which used exclusively NPR data, included 1235 patients from 1987 to 2017, our study combined SPR and NPR data reports of 1035 patients from 1991 to 2018. In the Esposito study, 186 malignancies of all causes were identified among patients with acromegaly compared with 144 expected in the general population (SIR 1.3; 95% CI 1.1-1.5). In the present study, cancer of all causes was diagnosed in 16.2% (n = 168) of the patients with acromegaly and in 13.2% (n = 1358) of matched controls. The adjusted HR (1.26; 1.07-1.48) for cancer overall in the present study was consistent with the findings reported by Esposito et al (40). However, an estimated SIR replaces matched controls with population-level statistics and does not incorporate individual-level covariates. This methodological difference explains the disparities in risk estimates observed between the 2 studies.

The risk for colorectal cancer has been debated for years (43). In our study, a higher colorectal cancer incidence was already seen in patients with an acromegaly diagnosis compared to the controls, supporting the importance of increased vigilance for colorectal symptoms at diagnosis and onward.

Lung cancer was also more common in patients with acromegaly in our study. The increased incidence was first noted more than 10 years after acromegaly diagnosis. In contrast, a study from the UK of patients with acromegaly reported a particularly low incidence of carcinoma of the bronchus (25). The authors speculated that environmental factors such as smoking habits and exposure to pollution and ionizing radiation may have been different in the acromegaly cohort compared to the general population. In our study, we do not have information about environmental factors. One reason for a higher incidence of lung cancer might be underlying chronic obstructive pulmonary diseases, for which smoking is a risk factor. However, because only one patient and one control subject had a diagnosis of chronic obstructive pulmonary disease at baseline, this explanation appears unlikely. Notably, 5 of our patients were diagnosed with malignant neuroendocrine pulmonary neoplasia, which may reflect undiagnosed hereditary endocrine cancer syndromes.

We also found an increased incidence of hematological cancer in patients with acromegaly. Our observation is in line with a recent meta-analysis of 19 studies including 11 494 patients where the authors found an increased incidence of malignant lymphoma, leukemia, and multiple myeloma (44).

Our observations from 5 years before the diagnosis of acromegaly showed a significantly increased adjusted HR for breast cancer in women, which is in line with a previous Swedish study that found an observed:expected ratio of 11:2.6 for breast cancer (45). A recently published study of a mouse model of acromegaly suggested that excess GH possesses a strong potential for promoting triple-negative breast cancer cell growth and metastasis (46); however, to our knowledge, this has not been studied in humans. We did not have the opportunity to further investigate the subtypes of breast cancer.

The number of individuals with thyroid cancer was low, including 2 patients and 4 controls: there were too few events to allow HR calculation, but the relative incidence appeared higher in the patient group. A higher incidence of thyroid cancer has been reported in a multinational study from Italy of 1512 patients with acromegaly (19) and in 2 recently published review articles (30, 44). Possible explanations are that acromegaly causes goiter and that frequent thyroid ultrasonography in patients with palpable thyroid nodules may lead to a higher diagnosis of some silent thyroid cancers including smaller papillary thyroid cancers (44). In Sweden, most endocrinologists do not perform routine thyroid ultrasound in patients with acromegaly.

Skin cancer was the only cancer type that showed a tendency toward a lower adjusted HR in the patients with acromegaly. Increased skin thickness, which may modulate skin cancer risk, has been described in a study using dermatoscopy and ultra-high-frequency ultrasound in patients with acromegaly (47).

Patients whose acromegaly was not biochemically controlled had a higher mortality rate compared to controls, as well as compared to patients who were biochemically controlled. This is in accordance with an earlier study by our group using the SPR where patients with biochemically controlled acromegaly had a normal life expectancy (2) and other studies that showed that normalization of GH and IGF-1 levels in patients with acromegaly normalize the mortality rate compared to the general population (9, 17, 25).

In our study, the mortality rate was higher in patients with cardiovascular disease but not in patients with diabetes, hypertension, or cerebrovascular disease. Other studies in patients with acromegaly have found a correlation between mortality and vascular or circulatory diseases (29, 48). Cancer-related mortality was not increased in the whole study population compared to controls in our study, which contrasts with some other studies (17, 29, 48) but agrees with a recent publication from the UK Acromegaly Register (49). We found cancer-specific death to be significantly increased only in the group who were 40 to 60 years of age. The fact that most patients were middle-aged at diagnosis of acromegaly in combination with the fact that cancer is uncommon at younger ages may have contributed to this finding. Not unexpectedly, cancer and other comorbidities such as cardiovascular diseases dominate as the main causes of death in older individuals. Patients may have a competing risk of death from cardiovascular disease or cancer, and, importantly, in cancer survivors the main cause of death is cardiovascular disease. Consequently, increased mortality due to cancer may not be ruled out, given the considerable uncertainty and wide CIs. Furthermore, in Sweden and other countries, there is a general trend toward a shift in major causes of death from cardiovascular disease to cancer-related mortality in the general population, although the pattern varies between different countries and regions.

A strength of the current study is its size; it is 1 of the largest cohort studies assessing cancer incidence and mortality risk in acromegaly and comparison to a matched control group. We have also assessed data from both the national registers, SPR and NPR, and patients registered in only 1 register with a diagnosis of acromegaly have been manually validated (3). Most of the patients in the cohort were reported in the SPR, which included long-term follow-up that further strengthens correct diagnosis and provides data on pituitary radiology, acromegaly treatments, biochemical control, and outcome.

A limitation is that actual serum GH and IGF-1 concentrations were not included in the SPR from the start. However, the biochemical laboratory reports were evaluated by experienced endocrinologists who considered patients biochemically controlled in the case of IGF-I level below the upper normal range for age and, vice versa, not controlled if above that level. Another limitation is that data regarding smoking as well as socioeconomic and environmental factors, which could influence some cancer types, were lacking.

In conclusion, several types of cancer were more common in patients with acromegaly compared to matched controls, which was seen even years before the diagnosis of acromegaly. Persistent elevated IGF-1 was not significantly associated with an increased adjusted HR for cancer, indicating an effect beyond GH hypersecretion. Among patients with acromegaly, the cancer-specific mortality rate was increased in those 40 to 60 years of age, while mortality due to other causes than cancer was increased further in all age groups if not biochemically controlled. Our findings support attention to symptoms of specific cancers immediately after the diagnosis of acromegaly and also call for patients to participate in national cancer screening programs.

## Data Availability

Available from the corresponding author on reasonable request.

## Abbreviations

CI: confidence interval
HR: hazard ratio
ICD: International Classification of Diseases
ICD-9: International Classification of Diseases, Ninth Revision
ICD-10: International Classification of Diseases, Tenth Revision
NPR: National Patient Register
SIR: Standardized Incidence Ratio
SPR: Swedish Pituitary Register

## References

[1] Aagaard C, Christophersen AS, Finnerup S, et al The prevalence of acromegaly is higher than previously reported: changes over a three-decade period. Clin Endocrinol (Oxf). 2022;97(6):773‐782. 36163677 10.1111/cen.14828 PMC9827885

[2] Arnardóttir S, Järås J, Burman P, et al Long-term outcomes of patients with acromegaly: a report from the Swedish pituitary register. Eur J Endocrinol. 2022;186(3):329‐339. 35007208 10.1530/EJE-21-0729

[3] Robert J, Tsatsaris E, Berinder K, et al Establishing a valid cohort of patients with acromegaly by combining the national patient register with the Swedish pituitary register. J Endocrinol Invest. 2024;47(4):995‐1003. 37851314 10.1007/s40618-023-02217-x PMC10965642

[4] Lavrentaki A, Paluzzi A, Wass JA, Karavitaki N. Epidemiology of acromegaly: review of population studies. Pituitary. 2017;20(1):4‐9. 27743174 10.1007/s11102-016-0754-x PMC5334410

[5] Melmed S . Medical progress: acromegaly. N Engl J Med. 2006;355(24):2558‐2573. 17167139 10.1056/NEJMra062453

[6] Ben-Shlomo A, Melmed S. Acromegaly. Endocrinol Metab Clin North Am. 2008;37(1):101‐122. 18226732 10.1016/j.ecl.2007.10.002 PMC2697616

[7] Esposito D, Olsson DS, Franzén S, et al Effect of diabetes on morbidity and mortality in patients with acromegaly. J Clin Endocrinol Metab. 2022;107(9):2483‐2492. 35779017 10.1210/clinem/dgac400 PMC9387713

[8] Vouzouneraki K, Franklin KA, Forsgren M, et al Temporal relationship of sleep apnea and acromegaly: a nationwide study. Endocrine. 2018;62(2):456‐463. 30066288 10.1007/s12020-018-1694-1 PMC6208862

[9] Holdaway IM . Excess mortality in acromegaly. Horm Res. 2007;68(Suppl 5):166‐172. 18174739 10.1159/000110617

[10] Dekkers OM, Biermasz NR, Pereira AM, Romijn JA, Vandenbroucke JP. Mortality in acromegaly: a metaanalysis. J Clin Endocrinol Metab. 2008;93(1):61‐67. 17971431 10.1210/jc.2007-1191

[11] Holdaway IM, Rajasoorya RC, Gamble GD. Factors influencing mortality in acromegaly. J Clin Endocrinol Metab. 2004;89(2):667‐674. 14764779 10.1210/jc.2003-031199

[12] Renehan AG, Zwahlen M, Minder C, O'Dwyer ST, Shalet SM, Egger M. Insulin-like growth factor (IGF)-I, IGF binding protein-3, and cancer risk: systematic review and meta-regression analysis. Lancet. 2004;363(9418):1346‐1353. 15110491 10.1016/S0140-6736(04)16044-3

[13] Clayton PE, Banerjee I, Murray PG, Renehan AG. Growth hormone, the insulin-like growth factor axis, insulin and cancer risk. Nat Rev Endocrinol. 2011;7(1):11‐24. 20956999 10.1038/nrendo.2010.171

[14] Dal J, Leisner MZ, Hermansen K, et al Cancer incidence in patients with acromegaly: a cohort study and meta-analysis of the literature. J Clin Endocrinol Metab. 2018;103(6):2182‐2188. 29590449 10.1210/jc.2017-02457

[15] Falch CM, Olarescu NC, Bollerslev J, Dekkers OM, Heck A. Trends in incidence and mortality risk for acromegaly in Norway: a cohort study. Endocrine. 2023;80(1):152‐159. 36525222 10.1007/s12020-022-03275-6 PMC10060282

[16] Kauppinen-Mäkelin R, Sane T, Reunanen A, et al A nationwide survey of mortality in acromegaly. J Clin Endocrinol Metab. 2005;90(7):4081‐4086. 15886256 10.1210/jc.2004-1381

[17] Mercado M, Gonzalez B, Vargas G, et al Successful mortality reduction and control of comorbidities in patients with acromegaly followed at a highly specialized multidisciplinary clinic. J Clin Endocrinol Metab. 2014;99(12):4438‐4446. 25210882 10.1210/jc.2014-2670

[18] Rokkas T, Pistiolas D, Sechopoulos P, Margantinis G, Koukoulis G. Risk of colorectal neoplasm in patients with acromegaly: a meta-analysis. World J Gastroenterol. 2008;14(22):3484‐3489. 18567075 10.3748/wjg.14.3484 PMC2716609

[19] Terzolo M, Reimondo G, Berchialla P, et al Acromegaly is associated with increased cancer risk: a survey in Italy. Endocr Relat Cancer. 2017;24(9):495‐504. 28710115 10.1530/ERC-16-0553

[20] Gasperi M, Martino E, Manetti L, et al Prevalence of thyroid diseases in patients with acromegaly: results of an Italian multi-center study. J Endocrinol Invest. 2002;25(3):240‐245. 11936466 10.1007/BF03343997

[21] Wolinski K, Czarnywojtek A, Ruchala M. Risk of thyroid nodular disease and thyroid cancer in patients with acromegaly: meta-analysis and systematic review. PLoS One. 2014;9(2):e88787. 24551163 10.1371/journal.pone.0088787 PMC3925168

[22] Boguszewski CL, Ayuk J. Acromegaly and cancer: an old debate revisited. Eur J Endocrinol. 2016;175(4):R147‐R156. 27089890 10.1530/EJE-16-0178

[23] Mestron A, Webb SM, Astorga R, et al Epidemiology, clinical characteristics, outcome, morbidity and mortality in acromegaly based on the Spanish acromegaly registry (Registro Espanol de Acromegalia, REA). Eur J Endocrinol. 2004;151(4):439‐446. 15476442 10.1530/eje.0.1510439

[24] Watts EL, Goldacre R, Key TJ, Allen NE, Travis RC, Perez-Cornago A. Hormone-related diseases and prostate cancer: an English national record linkage study. Int J Cancer. 2020;147(3):803‐810. 31755099 10.1002/ijc.32808 PMC7318262

[25] Orme SM, McNally RJ, Cartwright RA, Belchetz PE. Mortality and cancer incidence in acromegaly: a retrospective cohort study. United Kingdom Acromegaly Study Group. J Clin Endocrinol Metab. 1998;83(8):2730‐2734. 9709939 10.1210/jcem.83.8.5007

[26] Petroff D, Tönjes A, Grussendorf M, et al The incidence of cancer among acromegaly patients: results from the German acromegaly registry. J Clin Endocrinol Metab. 2015;100(10):3894‐3902. 26244491 10.1210/jc.2015-2372

[27] Renehan AG, O'Connell J, O'Halloran D, et al Acromegaly and colorectal cancer: a comprehensive review of epidemiology, biological mechanisms, and clinical implications. Horm Metab Res. 2003;35(11/12):712‐725. 14710350 10.1055/s-2004-814150

[28] Dal J, Feldt-Rasmussen U, Andersen M, et al Acromegaly incidence, prevalence, complications and long-term prognosis: a nationwide cohort study. Eur J Endocrinol. 2016;175(3):181‐190. 27280374 10.1530/EJE-16-0117

[29] Esposito D, Ragnarsson O, Granfeldt D, Marlow T, Johannsson G, Olsson DS. Decreasing mortality and changes in treatment patterns in patients with acromegaly from a nationwide study. Eur J Endocrinol. 2018;178(5):459‐469. 29483205 10.1530/EJE-18-0015

[30] Danilowicz K, Sosa S. Acromegaly and cancer: an update. Arch Med Res. 2023;54(8):102914. 38007382 10.1016/j.arcmed.2023.102914

[31] Barlow L, Westergren K, Holmberg L, Talbäck M. The completeness of the Swedish cancer register: a sample survey for year 1998. Acta Oncol. 2009;48(1):27‐33. 18767000 10.1080/02841860802247664

[32] Esposito D, Ragnarsson O, Johannsson G, Olsson DS. Prolonged diagnostic delay in acromegaly is associated with increased morbidity and mortality. Eur J Endocrinol. 2020;182(6):523‐531. 32213651 10.1530/EJE-20-0019

[33] Forsgren M, Dahlgren C, Alkebro C, et al Estimating diagnostic delay in patients with pituitary adenomas in Sweden: a cross-sectional study. BMJ Open. 2025;15(6):e097804. 10.1136/bmjopen-2024-097804 PMC12186051 40550723

[34] Freda PU, Bruce JN, Jin Z, et al Prospective, longitudinal study of cancer predictors and rates in a New York City cohort of 598 patients with acromegaly. J Clin Endocrinol Metab. 2025;110(5):1247‐1257. 38986012 10.1210/clinem/dgae469 PMC12168067

[35] Pekic S, Stojanovic M, Popovic V. Pituitary tumors and the risk of other malignancies: is the relationship coincidental or causal? Endocr Oncol. 2022;2(1):R1‐R13. 37435457 10.1530/EO-21-0033 PMC10259320

[36] Loeper S, Ezzat S. Acromegaly: re-thinking the cancer risk. Rev Endocr Metab Disord. 2008;9(1):41‐58. 18157698 10.1007/s11154-007-9063-z

[37] Gadelha MR, Kasuki L, Korbonits M. The genetic background of acromegaly. Pituitary. 2017;20(1):10‐21. 28161730 10.1007/s11102-017-0789-7 PMC5334425

[38] Vasilev V, Daly AF, Zacharieva S, Beckers A. Clinical and molecular update on genetic causes of pituitary adenomas. Horm Metab Res. 2020;52(08):553‐561. 32299111 10.1055/a-1143-5930

[39] Oguz SH, Firlatan B, Sendur SN, Dagdelen S, Erbas T. Follow, consider, and catch: second primary tumors in acromegaly patients. Endocrine. 2023;80(1):160‐173. 36517649 10.1007/s12020-022-03282-7

[40] Esposito D, Ragnarsson O, Johannsson G, Olsson DS. Incidence of benign and malignant tumors in patients with acromegaly is increased: a nationwide population-based study. J Clin Endocrinol Metab. 2021;106(12):3487‐3496. 34343297 10.1210/clinem/dgab560

[41] Kim YS, Yun JS, Kim H, et al Acromegaly and the risk of cancer: a nationwide population-based cohort study in Korea. Eur J Endocrinol. 2025;192(3):220‐227. 40037898 10.1093/ejendo/lvaf029

[42] Duskin-Bitan H, Perez A, Netzer D, et al Increased cancer risk in a cohort of patients with acromegaly in Israel. Endocr Relat Cancer. 2025;32(5):e240087. 40062775 10.1530/ERC-24-0087

[43] Kasuki L, Maia B, Gadelha MR. Acromegaly and colorectal neoplasm: an update. Front Endocrinol (Lausanne). 2022;13:924952. 35795151 10.3389/fendo.2022.924952 PMC9251006

[44] Xiao Z, Xiao P, Wang Y, Fang C, Li Y. Risk of cancer in acromegaly patients: an updated meta-analysis and systematic review. PLoS One. 2023;18(11):e0285335. 38032888 10.1371/journal.pone.0285335 PMC10688666

[45] Bengtsson BA, Edén S, Ernest I, Odén A, Sjögren B. Epidemiology and long-term survival in acromegaly. A study of 166 cases diagnosed between 1955 and 1984. Acta Med Scand. 1988;223(4):327‐335. 3369313 10.1111/j.0954-6820.1988.tb15881.x

[46] Kang CW, Oh JH, Wang EK, et al Excess endocrine growth hormone in acromegaly promotes the aggressiveness and metastasis of triple-negative breast cancer. iScience. 2024;27(7):110137. 39006481 10.1016/j.isci.2024.110137 PMC11246000

[47] Guo X, Wang Y, Yao Y, et al Sub-macroscopic skin presentation of acromegaly and effect of pituitary tumor surgery: a study using dermatoscopy and ultra-high-frequency ultrasound. Front Endocrinol (Lausanne). 2022;13:1093942. 36818464 10.3389/fendo.2022.1093942 PMC9933496

[48] Arosio M, Reimondo G, Malchiodi E, et al Predictors of morbidity and mortality in acromegaly: an Italian survey. Eur J Endocrinol. 2012;167(2):189‐198. 22596288 10.1530/EJE-12-0084

[49] Orme S, McNally R, James PW, et al Increased mortality in acromegaly is due to vascular and respiratory disease and is normalised by control of GH levels: retrospective analysis from the UK acromegaly register 1970-2016. Clin Endocrinol (Oxf). 2024;100(6):558‐564. 38652736 10.1111/cen.15060

